# How to Introduce a Gutta-Percha Filling

**Published:** 1890-12

**Authors:** Merit Wells

**Affiliations:** Indianapolis.


AMERICAN JOURNAL

—OF—

DENTAL SCIENCE.

VOL. XXIV. BALTIMORE, DECEMBER, 1890. No. 8.

ARTICLE I.

### HOW TO INTRODUCE A GUTTA-PERCHA FILLING.

BY DR. MERIT WELLS, INDIANAPOLIS.

We suppose first that the cavity has been properly prepared, protected and dried. First, take a piece of gutta-percha a little larger than will be required to fill the cavity. Warm gradually and shape it into a pellet or bean shape, being careful not to burn the gum. Use dry heat (on a heated slab or by passing rapidly back and forth through the flame of a lamp). When the gutta-percha is pliable, take a smooth spatula or blunt instrument and press the mass into the cavity till it is too full. If the gutta-percha seems to be unyielding before the cavity is full, heat the instrument slightly and apply till the gutta-percha becomes pliable. When the cavity is full allow the filling to cool, then heat the instrument and press near the center of the filling. Press the instrument away from the center down to the surface of the opening and off, carrying away the surplus. Then allow the gutta-percha to cool and again heat the instrument and press in the opposite side of the cavity similarly. Cool again and thus treat the whole surface of the filling. Avoid pressing backward and forward, as thus you get the filling to playing or rocking in the cavity. In case the nerve is exposed or nearly so, shape enough material to fill the whole cavity in such a way that a rounded, smooth point may be brought in contact with the exposed nerve or sensitive point. The cavity being all ready (dry, etc.), heat the point of gutta-percha which is intended to touch the nerve or sensitive point till it is pliable, then press the surface against a smooth surface, making the gutta-percha surface smooth and cool at the same time. Then press the gutta-percha to place quickly and gently till the bottom is reached, leaving no space; then rest till the material has cooled, then proceed to fill as described before, being careful to keep the gutta-percha as cool as possible to work, as the heat is liable to cause pain and inflammation in the pulp. When the patient winces at the first touch of the gutta-percha at the bottom of the cavity, you may know you have touched the nerve, and no more pressure should then be made till the mass cools, else you might cause the gutta-percha to press in upon the pulp and thus cause pain and failure.

In filling roots press in place the softened mass large enough to fill the pulp chamber. Then with smooth broach heated, press through the gutta-percha into the root; rotate the instrument and withdraw, and insert again rapidly; each thrust carrying on the side of the broach a portion of the gutta-percha into the root. Heat the broach as it gets cool and thrust again till the root is full. The advantage of the large mass at first is that it holds to the side of the cavity and prevents the instrument from pulling the gutta-percha out of the root as you withdraw it. The gutta-percha tends to hold to the heated instrument more than to the walls of the cavity. But the surface of the walls of the cavity being greater than that of the instrument, the latter may be rotated and withdrawn, leaving the gutta-percha in the root.

DISCUSSION.

Dr. Chappell. On the subject of filling teeth with gutta-percha, the first point to consider is the necessity of filling with gutta-percha. The doctor only spoke of filling teeth with gutta-percha, irrespective of the conditions. To know when to fill teeth with gutta-percha, I regard as one of the most important questions.

Dr. Wells. I only spoke of how to introduce it.

Dr. Chappell. I speak in regard to gutta-percha fillings. It is always best first to consider when it should be introduced. Of course, the question of how to introduce it is another matter. To confine the subject to how to introduce the filling, of course, would make it rather shorter. I should burnish my gutta-percha with a warm instrument, and also the surface of the filling after I have properly dried it and given antiseptic treatment, and use the warm instrument until it becomes plastic. Burnish the gutta-percha against the walls of the cavity so as to form a lining, and then introduce my particles smaller than you do, from the fact that I wish to have all the air excluded and wish to know that each particle that I place in the cavity goes to its place. In filling with gutta-percha, like when we bridge the filling with gold, we know that in the use of it the gutta-percha sometimes is not properly prepared. If we place a great quantity of gutta-percha in a tooth it has a tendency in a short time to bulge; that is one trouble with it. If the filling is soft the gutta-percha will puff out, so that it becomes necessary for us to use top dressing. The best thing that I have used to secure the walls with is adhesive gutta-percha. We can frequently take the gutta-percha out of a tooth and find that the walls there are disintegrated. If we will examine the filling of the tooth still further we will find that the filling is bulging, and that there is more or less recession or withdrawing from the walls of the cavity. To keep the gutta-percha filling in good shape depends on the manufacture; that is, it depends on the quality of the gutta-percha. In filling roots of teeth I have quit filling the pulp chamber proper with gutta-percha and then putting on plastic filling on top of that, from the fact that I think I get better results by going further down with oxyphosphate or even with alloy. I desire to have the alloy placed down low enough so that there will be an anchorage by making a crown of that character, by making an anchorage in the pulp cavity, and if it is a gold filling I prefer to go lower down and make the anchorage with oxyphosphate or with gold, on account of the difficulty of bulging. The earthy matter that is introduced into the gutta-percha holds because it will hold its volume. A great deal of our gutta-percha that we have now is so adulterated with a substance near akin to gutta-percha; I forget the name now, but it has entered into the composition of our gutta-percha work. I think Dr. Caulk is making about the best gutta-percha that we are using. I had some of Dr. Flag's at one time; it was hard cement and hard gutta-percha, and seemed to me the best thing I ever found, but as a reliable gutta-percha I think Caulk's seems to give the best satisfaction. I think it was the diamond cement. It was incorporated into the other substances. I think for temporary filling there is nothing better than gutta-percha, and I fill with it almost exclusively.

It is a very hard matter for me to say anything about gutta-percha fillings unless we discuss the condition of the tooth, and the absolute necessity for the use of gutta-percha. As to the application of hot gutta-percha over an inflamed pulp, or rather open pulp, I introduce the gutta-percha very close, if not in actual contact, not only with the fibril life, but with the pulp, as a condition itself without much danger of pain. But they must be thoroughly dry and in good shape. There must be no pressure upon the nerve; if there is we will have pain. There is a vascularity about the walls of a pulp chamber that we hardly appreciate sometimes when we are placing a filling; that is, we are so near to that membrane that while there is an ossiffic deposit over it so that the dentine will press down, there will be some ache about it to a certain extent, and the fibrilla there are so close in contact with the pulp that they will take on irritation. Gutta-percha, of course, in such case is the best substance we can possibly use, on account of its being nearer the normal condition than any metal. As a general filling in a perfect tooth, and without irritability, gutta-percha, I think, is the best filling we can use. Each operator, of course, has his own peculiar ways of introducing a filling for expedition and convenience.

Dr. Frank Overholser. I would like to ask Dr. Wells what method he uses in heating gutta-percha?

Dr. Wells. Pass it through the flame of a lamp.

Dr. Overholser. Do you find that it sticks to your pliers very much and makes it hard work?

Dr. Wells. No, sir.

Dr. Overholser. That seems to be the greatest difficulty I have with gutta-percha. It sticks to everything that we touch, and sticks to my pliers.

Dr. Wells. I do not think it is necessary to get the whole mass soft. Turn it over as it comes in contact with the cavity, and becomes adapted to the walls of the cavity. If the center of it is not soft it will not make any difference. That will press the soft part into shape, and it cools so much the sooner. The larger the mass you have, the more heat there is in it that must be dissipated. Sometimes in filling, when the center part is cooled and the surface soft, you press that center which is hard, against the softened surface as it becomes fitted to the wall, and it will cool quicker. There is one trouble; you have so much heat in there that it produces pain, and it will continue until it cools off. When I go to fill on an exposed pulp, I use a piece of gutta-percha that is larger than where the pulp is exposed, and I heat and press it upon a surface that is smooth, rotating it so as to give it a kind of rounded surface, and at the same time that it is being rounded and smoothed it is being cooled, and that surface is cooler than the mass underneath, and as I bring it over the pulp it is still pliable enough from the heat from the center, and as I press it down it will fit the walls, and is less liable to pass through into the surface of the pulp than if that surface was all very soft, so that in passing it through the flame of a lamp, I do not care about the whole of the mass being soft. I think that the surface that comes in contact with the walls of the cavity is pliable, and that is all I want, and I press it into place. My notion is that the more you begin to work it around, first one way and then the other, the more liable you are to loosen the filling. I take the mass large enough, and press it in, so that when it gets in, the diameter of the gutta-percha is less than that of the cavity, so that it strikes the bottom and excludes the air by pressing from the bottom of the cavity up to the walls at the top, getting the holes full, getting cool, and never cooler than the wall. In pressing the instrument down, every time you press down, press towards the wall, and not fill up from the wall.

Dr. Wilson. I use gutta-percha to a great extent just as Dr. Wells does, only that on going to fill a very large cavity with gutta-percha, I do not always use one piece, but if I am going to fill a small cavity, I always use one piece. His idea of pushing your instrument always towards the edge of the cavity, I believe, is correct. I notice in discussing this subject, in different societies, that some men claim that you cannot put in gutta-percha filling without moisture getting into the filling. I do not doubt but what that may be true, but I do know that gutta percha can be put in a tooth so that the secretions of the mouth will not get in between the filling and the tooth. I do not say that I always do that, but I always aim to, and if I do not, I think it is my fault, and not the fault of the guttapercha. I think the difficulty of working has something to do with the filling. I use the least little bit of chlora percha very often before I warm the filling that I am to fill the cavity with, and I use this gutta-percha very often in children's teeth, instead of using cement. I think it will last longer than almost anything else you can use as a temporary filling for children's teeth. I use it not because it looks better, because I do not think it looks as well as phosphate, but I use it because I think, and in fact I know, that it will last longer in my hands. With chlora-percha, I think, of course, if the pulp was exposed I would hardly want to use that, but I think it enables you to get the actual contact between the walls of the cavity and your gutta-percha.

Some one said that he was bothered by his instrument sticking. Sometimes I use a little soapstone for that. Just the least mite will keep the instrument from sticking to the gutta percha, and will enable you to get along a little better. In root filling I do not use quite as much chlora percha as I used to. I use the kinds that can be bought at the dental depots, or have my assistant make them, and I do not aim to use near as much gutta-percha in the root as I used to; another reason, I think sometimes when we use chlora percha, and then use gutta-percha, you have not got the color; you color your tooth red, and do not get as good a looking tooth after it is filled as if you had used even oxyphosphate. There is a tendency to leave in the tooth next to the gum a little color that chlora percha has produced by showing through the tooth.

Dr. Wells. Do you use colored gutta-percha?

Dr. Wilson. Yes, I use the red; that is what I was talking about.

Dr. Overholser. I would like to ask Dr. Wells what he understands is the cause of this gutta-percha bulging so. Is it a tendency to ball up into a sphere, as if inherent in the gutta-percha, or is it from the earthly compound that enters into it?

Dr. Wells. I could not explain that. I must say that in the observation of fillings that I have inserted, I have not seen much tendency of the gutta-percha to ball; that is; I mean to raise out of the cavity, I have always, or as a general rule, used the old-fashioned Hill's stopping. I have tried a few other preparations, and in many I find a tendency, as Dr. Overholser says, to stick. Just as soon as you get them soft they stick to the instrument, and when you put it in the cavity and take the instrument away the gutta-percha comes away with the instrument. But with Hill's stopping I have never observed much of that tendency to swell out; and even if I had observed it, I could not tell the reason why it should do so. I am sorry I can not answer the Doctor's question in regard to that.

Dr. Oliver. I think that in almost every case you will find the bulging is caused by the gutta-percha being too warm, and it bulges when it cools.

Dr. Hunt. Dr. Chappell suggests that in cooling it contracts.

Dr. Oliver. It depends a good deal on the process of hardening it.

Dr. Hunt. As Dr. Chappell made the suggestion about bulging, I would like to have his opinion as to what he thinks is the cause of it.

Dr. Chappell. There is much of our gutta-percha adulterated. I mean that instead of using the earthy matter they use this preparation in place of the gutta-percha. The bulging is the deterioration of the material of the filling itself, on account of not being pure gutta-percha. I have never had as much trouble with Hill's stopping or pulp cement. I have found a little difficulty in the warming as the cause, but there is one stopping made by Dr. Caulk that is as near being the nicest thing in the world as anything I ever saw. He makes several grades, from very hard to reasonably soft.

Dr. Hunt. It is a lost art.

Dr. Chappell. It is a lost art, and a great many other arts will go with it.

Dr. Oliver. Is it through any fault in manipulating the gutta percha, or that the gutta-percha is so porous that it will absorb so as to alter the filling materially?

Dr. Wells. I believe that there are some agents which will produce the destruction chemically of the gutta-percha. I believe that in some of them that agent is absent. I have taken gutta-percha out of cavities that have been filled more than a year, and found no unusual odor. Of course, if we burn the gutta-percha it will decompose, and you will have a very sensible odor. I think, also, that whisky has a tendency to decompose it. I received that idea from an old scientific gentleman. I filled a tooth for him with gutta-percha. It was on the posterior surface of a molar or bicuspid, I am not sure which, and it was a tooth that I thought would not stand the pressure. It was loose, and yet he wanted to save the tooth, and it appeared to me that that was the proper thing to use. After he had used it awhile he said to me that he wanted me to remove it, fill it, and put in something that did not smell so bad as gutta-percha. He said the whisky seemed to destroy the gutta-percha and soften it, and it smelled bad. I believe he was right. I believe that there is chemical action of the liquor on the gutta-percha. There may be other things besides whisky that will destroy it.

A Member. How did it get up into the root?

Dr. Hunt. It was not the root; it was the surface.

Dr. Wells. Of course, if you use a filling of any kind on a root, you may expect to have pressure on the root, regardless of the filling material. There is a very bad odor from a decomposing gutta-percha filling, and when that is the case I do not know what causes it, unless it is from whisky or some other agent. Of course, there must be some cause. I have known fillings that have been in six or eight, or ten years. One lady said a filling had been in seven years and it was good but worn some. Now, of course, if the gutta-percha was going to deteriorate, caused by the presence of moisture and heat, they would have been decomposed long ago, so that I could not tell only as I got the idea from this rather scientific gentleman who said that whisky destroyed the gutta-percha and made it smell bad.

Dr. Burt. I would like to ask Dr. Wells how long he has been filling roots that way?

Dr. Wells. I do not know that I can tell exactly. I would think as much as fifteen or twenty years.

Dr. Burt. Did you ever take any teeth out afterward that had been filled with gutta-percha?

Dr. Wells. I do not remember that I ever did.

Dr. Burt. I wanted to know the condition of it; that was all. I do not fill roots that way.

Dr. Morse. We are taught that if we wish to avoid pain we must avoid pressure on the exposed pulp; and to insert gutta-percha even as warm as the pulp or as it possibly would stand it, it would require more or less pressure, I should think, on the pulp, and therefore cause more or less pain. While the material in itself, I should think, would be an excellent thing to act as a non-conductor between the metallic filling and the exposed pulp, or pulp nearly exposed, it seems to me that it would be a very difficult matter to apply the gutta-percha over the pulp without causing pressure, because it has to be put in with a certain amount of pressure, and you have it attached to all the walls and leave no air space over the pulp, and it requires a very delicate manipulation, certainly. While I never practiced it and have not tried it, it would appear very difficult work for me, at least. I have noticed the decomposition of gutta-percha spoken of, but most usually it seems to me there was more or less leakage to the filling in those cases.

Dr. Chappell. Decomposition of filling, if you will observe, is in teeth that have no occluding tooth or teeth and that are not kept clean; you will find in those cases a tendency to bulging. By means of considerable friction and washing you will find that the filling does not bulge, but there will be gradual attrition there, so as to get it off. As the surface deteriorates it is gradually worn off. I have teeth that have been filled for some of my patients for the last six or eight years with gutta-percha, and I have to fill them every few years for the simple reason that they will not consent to have gold put in them, and I would not consent to put in amalgam or anything else, so they have kept them in that way and they gradually wear out. There is no tendency at all in those teeth to disintegrate. If you use pure gutta-percha and a sufficient amount of foreign inorganic matter so as to make it solid, you will not have that trouble of bulging. But there is a disposition on the part of a great many of our manufacturers to make it with as great profit as possible. I have observed one fact about gutta-percha fillings, and that is that you must keep the surface clean. I think that the whisky case mentioned came from the simple fact that there is a great variety of secretions in the mouth around the teeth and filling, and, of course, it does not absorb to a great extent the secretions of the mouth, but they will disintegrate the rubber unless it is vulcanized with a certain degree of heat. If you had any old rubber hose or anything of that kind where the degree of vulcanization is only to such extent as the whole mass can hold it in form and give it pliability, the rubber will disintegrate very rapidly; but if you have a sufficient amount of heat and vulcanize it to a certain extent, so as to throw it off and leave the inorganic matter there, it will become a much more difficult matter for you to disintegrate the rubber or the gutta-percha. You will find that to be the rule. Rubber, of course, can be made with a higher degree of perfection than the gutta-percha in the arts, and you will find that the same way in filling teeth, that it will discolor unless certain conditions are constantly obtained.

The President. I would like to ask the Doctor if you ever find, under gutta-percha filling that you put in where the pulp is exposed, that the pulp was dead and gone?

Dr. Chappell. Yes, I often find the pulp dead without any decay. Why the pulp dies I do not know; I do not suppose the Angel Gabriel could tell.

The President. I merely asked the question because I supposed that in cases where the pulp is exposed pretty badly it has been killed.

Dr. Chappell. There is many a pulp dead where we fill a cavity when we think the pulp is alive, but there is no sign of a pulp there. It is simply a cavity and the foramen is closed.

Dr. Durr. I would like to ask any of the gentlemen if they ever use shellac and oxide of zinc for root filling or temporary stopping. I have used it a long time myself.

Dr. Wells. No, I have never used it.

Dr. Burt. I have used it somewhat myself. I use shellac rather thick, then mix the oxide of zinc with it and make a thick paste or putty, and you can incorporate carbolic acid with it, and it makes an excellent stopping. In either temporary fillings for children or root fillings you can insert it, and the moisture will not have any effect on it whatever.

Dr. Hunt. Does it become pretty hard?

Dr. Durr. Yes, but it is slow in hardening. It takes perhaps a couple of days to harden thoroughly. That is one objection to its use for temporary filling, but you can remove a portion and put cement in, in cases where the filling should be something that hardens very quickly; and also in teeth that are very sensitive you can dissolve it with the acid and insert it with the filling; the tooth has a tendency, of course, to become sensitive.

Dr. Wells. In closing the discussion it seems to me that it has been broadened a great deal, and I hope the gentlemen will excuse me for seeming to have neglected that branch. I did not propose in my paper to tell when or where you should use gutta-percha for filling, but merely how to introduce it to make it the most useful; to do it according to my notion in the easiest and best way; to put it in a cavity without regard to why it should be there; therefore, I left out all theories in regard to the cleansing of the cavity or the condition of the pulp, and all those pathological conditions. I did not intend to discuss whether it was a better filling than anything else, so that you will understand that I merely intended in my paper to tell how to introduce it. After you have determined to use it, then how to do it. That is one reason why I did not speak of many of these other things that Dr. Chappell and many others have mentioned Those are all proper things to consider, but inasmuch as my paper did not embrace that, I said nothing about it. In regard to some questions that have been asked me about shellac, I would say that while I have not used it I have heard it recommended by others. Dr. Leo, a nephew of Dr. Gardner and J. Oscar Bell, at the time our Association met with the Kentucky Association at Louisville, spoke of using that and recommended it.

Dr. Durr. I would like to ask if the gentleman used shellac the same way, mixed with the oxide of zinc, or shellac itself?

Dr. Wells. I think he used just the pure shellac.

Dr. Durr. I think you will find a difference. This makes a plastic paste that you can force into the smallest root canal, and the shellac will harden rather too quickly for that purpose.

Dr. Hunt. Do you use heat in introducing it ?

Dr. Durr. No; no heat whatever; just a plastic paste. You can make it of any consistency you want to, and I find it is an advantage to incorporate a little carbolic acid with it; it seems to make a better filling. I have removed fillings of that kind. I filled a tooth for a lady who broke off the crown of a lateral and I had occasion to put on another crown. I drilled up as far as I could drill, but I could not find any moisture or any offensive odor whatever.

Dr. Hunt. Do you think this makes a tight joint between them?

Dr. Durr. I think it does; I think it makes a water-tight joint.

Dr. Hunt. The process of hardening is caused by the evaporation of the alcohol, I suppose, is it?

Dr. Durr. Yes. The process of hardening is very slow; that is the principal objection in using it. I filled a deciduous tooth for my little girl that I think was in the cavity two years.

A Member. What was the color of the filling?

Dr. Durr. Something like gutta-percha; something of a peach red color.

The President. You can use a white shellac and make it pretty nearly white.

Dr. Durr. I presume you can. I never use it; I always use the other; I have been using it for several years.

Dr. Overholser. I wish to diverge just a little on the subject of root fillings. Remarks have been made regarding the capping of pulps. Two years ago I wrote a paper on the subject, in reference to where the pulp was found exposed or devitalized. I said very little about the capping of pulps, because it seemed to be the experience of the profession that the capping of pulps has been a failure. Now I considered that that was really a process that was almost obsolete in dentistry. That is a rather sweeping assertion, but at the same time I believed it to be the case. Aside from the very few cases of early childhood, I would like to hear the general expression as to when and where we should cap pulps. Owing to an unfortunate circumstance in the Dental Association two years ago when my paper was presented, the subject was not spoken of at all, but one of the remedies that I suggested seemed to get all the argument and nothing was said about when and where we should cap pulps. I should like to hear that subject discussed. Possibly you would like to know my position on the subject, and that is that I always devitalize the pulp; that is, I do for any one except a very young person, and I never cap a pulp unless I see it and know it is there. As Dr. Chappell said a moment ago, sometimes pulps are capped, or rather canals are capped. We ought not to cap anything until we know it is there, and if the pulp has not ached, then there seems to be all the more reason to find out whether it is there or not. Sometimes we inquire of the patient, "Has the tooth ached?" The patient says "No." Possibly the reason it has not ached is that the pulp has died out gradually, and it would never pain unless it was an abscessed tooth, and that is going on with a rather low degree of inflammation and not giving trouble.

Dr. Chappell. On the subject referred to by Dr. Overholser as to when a tooth has ached, I would like to ask whether it is pulp irritation, eroded enamel, denuding the dentine or removing the enamel slightly so that there is febrile irritation, or whether he has reference to pulpitis or abscess and so on.

Dr. Overholser. I might say pulpitis. Of course, there are a great many people that we can not do anything with, if there is abrasion of the enamel; people who for several years have a sensitive tooth that they can not eat on, and who can not take anything hot or cold into the mouth; and it is much more satisfactory to drill into the tooth and destroy the pulp proper and cap it and give the patient satisfaction, without any sentimentality.

Dr. Chappell. The reason why I ask the question is from the simple fact that there are so many persons that fail to consider the difference in individuals and the difference in the texture of the tooth. For instance, take a child in the process of development where the tubules of the teeth are large and the fibril are necessarily large. We will expose the pulp, and I will illustrate what I meant by exposure in this way; if this window and these doors were opened we would be exposed to the inclemency of the weather, but if we were to take off all our clothing and go out there we would be denuded and extremely exposed. That is an extravagant comparison, but it is frequently the case that the pulp is entirely in good order but the fibril life is in a state of irritation. We can not say that it is inflamed because it does not possess the elements or structure capable of what we know now as inflammation; but there is a sufficient amount of irritation there to produce pain at the touch or under pressure, and chemical erosion is going on and there is pain; the tooth has ached, when if we cleansed the tooth and dried it and dressed it and used gutta percha, we would have a good tooth and no necessity of extracting the pulp. We must bear in mind that we must draw the line somewhere between health and disease, and we must be very careful whenever we find a tooth that has sensation, to know the degree of cold that will affect it. As to the treatment of tooth pulp, that means the treatment of fibril, the treatment of the undeveloped structure of the tooth. If it were not so, nearly all our cases in filling children's teeth would be failures, because whenever we find that the aggressive element of decay is penetrating the pulp, an effort of nature is made in the opposite direction. Then we have the resistence of the deposit around there and if we can help nature in protecting that, we have done just what a good dentist ought to—help serve nature in its efforts.

But that is not what I ask about in regard to the condition when the tooth has ached. We must not always depend for our diagnosis on what a patient tells us. When there is any disease or trouble anywhere, we must diagnose by sensation, and find out whether the pulp is really affected or not, or whether it is the fibril. We must get a healthy tooth and see that it is all right and go on until we come to the diseased part. A great many have been led to believe, from some discussions in the societies, that whenever a tooth has a cavity that has the least bit of sensitiveness, they should destroy the nerve by making an application of arsenic or anything else that is appropriate. Before I do that, I see whether the pulp is alive and use other means to test that tooth as to whether the pulp is dead or alive.

Dr. Morse. I wish a little information on the point of nomenclature in regard to the capping of pulps. We hear about pulp capping and pulp-lining and I want to ask whether they mean that the pulp is exposed or not exposed, or whether there is a thin membrane or small portion of the tooth that covers the pulp. According to my understanding, capping a pulp would be only when it is exposed, and I wish to know whether that is correct or not.

Dr. Overholser. That is the kind of pulp that I had in my mind when I said an exposed pulp.

Dr Chappell. The doctor has reference to what is known as a denuded pulp, where there is lamina of the bone, as the Doctor suggested. That is what you had reference to—where the pulp is exposed—is it?

Dr. Morse. Yes.

Dr. Wells. Since we have got on another subject, I would say that I am astonished to hear that the term "filling over an exposed pulp" is obsolete. I know I have been doing it right along for twenty years or more—nearly thirty years. I think I have been filling over an exposed pulp every month in the year, and a good many of them some months, and a good many have died, while a good many have lived. I found them alive years afterward. I think I have had fifty teeth with the pulp exposed at a small point and a healthy condition. I mean without any inflammation or ulceration. We must not put any kind of filling over a pulp that is discharging pus, but if the structure is all there, covering the pulp and all the live tissues there except that bony lamina, or a portion of that, and you can get your gutta percha filling or something else if there is anything better (I do not know anything better)—if you can get that applied right down to that without pressing the pulp and without diminishing the size of the pulp, because when you press any part you diminish the size—if you can get that down and save one-quarter of those pulps for a year, and perhaps a good many years afterward, I think it is worth the trial even if you do find the tooth aching somewhat and you have to take out the filling and destroy the pulp. I have taken out a good many gutta-percha fillings and destroyed the pulp when there was inflammation, and I have saved them and many are alive and healthy yet.

The first thing that gave me a great impression with regard to the utility of gutta-percha over an exposed pulp was one in my own mouth. The doctor excavated some very large cavities. I did not know that there was any cavity in the tooth until by the appearance of it he detected signs of decay, made an opening, removed the decay, and the pulp was exposed. He put in a gutta-percha filling with Hill's Stopping and that remained until the next session of the Dental College. I returned and he removed that filling and put in a gold filling. That remained a good many years. Finally I lost that and I had it filled with a temporary filling of oxychloride of zinc, I think two or three times, and finally, after I had used it about fifteen years or more, the tooth became troublesome and sore. There was indication that the pulp was dead, and I had to remove the tooth finally, it became so troublesome. I took a file and commenced filing on the side of the root to see where the opening was, and as I filed down I saw evidence that there had been a deposit of substance where that pulp originally was, and I followed it down I think for a quarter of an inch from where I supposed the exposure commenced, or from the point where it was first exposed. I filed down there about a quarter of an inch before I found the place where the pulp had died. It just kept following the wall and receding until it went down about a quarter of an inch, and then there was a large surface protection a quarter of an inch thick, but why it had died I do not know. I think there might be a good many that would not die even after that long.

Dr. Bell. It seems to me that an exposed nerve, or pulp rather, is in the condition that there is not the least particle of dentine over it, in some one particular spot. It may be even as fine as the point of a needle, but when there is what we term "an exposed pulp," it seems to me that the dentine is entirely gone. When you have a very slight film of dentine over the pulp you would require there, it seems to me, something similar to a capping. It would have to be of a non-conducting substance, just the same as a capping. That would be termed an "intermediate." A capping would not be termed an "intermediate," because it would be directly upon the pulp, providing it was a substance that might press down in that way. There are various kinds of capping. You might use a metal capping and have that metal capping, first with some non-conducting substance over it, of a concave surface below and a convex surface above, and in that way you would protect the whole body. Now, I do not believe that all pulps ought to be capped that are exposed, nor I do not believe that all ought to be saved. I think that the general condition of the patient should be considered above everything. If the patient is enemic, particularly a woman having her family duties to look after, if the pulp of one of her teeth is entirely exposed and has given her some trouble, I think that under such circumstances it would be wise to devitalize. If the patient is young, strong, healthy, and having good recuperative power, I believe that even though the pulp has given some trouble it would be perfectly safe to cap it. But I govern myself in this way: I never fill directly after capping; I would cap the pulp and let the patient go for at least two weeks before I would proceed any further.

Dr. Hunt. In hearing the discussions here I was reminded of the period when I first learned of the capping of pulps. It came to me that we used to use goose quills for capping pulps, and it is a good non-conductor, splitting the quill and laying it in over the nerve as a protection. They did not work very well. Of course, we did not get very close contact with the goose quills, or if we did we had trouble. Then soon after that soapstone was used as a capping, with better results. Then it was at one time quite a practice or custom to fill gold in there, leaving a hollow in the gold, so as not to put any pressure on the center of the tooth that might produce pain from the pressure in contact with the nerve substance. All these discussions carry me back to the days when I first heard of capping pulps. As we have been digressing from the paper, I will state a little occurrence that took place at Louisville a good many years ago, along in the days of the first operations of filling pulp cavities. There was an elderly gentleman that had been in the practice of dentistry for a number of years, whose field of operations extended from New Orleans to Louisville, the gentleman practicing and stopping at Vicksburg, Memphis and Natchez. He was a Southern gentlemen, and really a very high type of a gentlemen. I was a very young dentist and called upon him, and in the course of the conversation I mentioned the fact that the roots of teeth were successfully filled. The old gentleman braced up and said yes, he knew that there were some young dentists that thought they had discovered something but they did not amount to anything. Well, having said that, I was considerably nettled and could not resist the temptation to fire back at him that there were also a great many fogies in the practice of dentistry that would not accept of an advance when it was presented to them. He got up and took me by the hand and said, "Come in the back room here, young man," and he opened a bottle of champagne.

Dr. Johnston. There is one thing that has not been referred to, and it occurs to me that there is something in it. I have had very little success in capping pulps after there has been an exposure and decay has reached such a point that it might become necessary to make a covering that would leave the pulp room. I do not think we will succeed from the fact that, if the pulp needs any room there, we have a ragged, uneven surface where the break is, that will continually irritate that pulp, and I do not think we can succeed. There are some few persons that will bear almost anything. We can do some things with some people that we can not do with others. Some people will tell you that the tooth never ached, but that it hurts a little. I believe that the pulp is continually irritated by the exposure of the broken, irregular surface, and it is unnecessary or impossible to save the tooth by capping. In all such cases I would devitalize the pulp.

Dr. Hunt. Do you think that in all pulps exposed to the bottom of the cavity there must necessarily be the ragged edge that you speak of? Do you not think that there is a protrusion of the pulp that would cause irritation?

Dr. Johnston. I think there is if the pulp is not possibly devitalized already.

Dr. Hunt. But in cases where a portion should be accidentally exposed and the pulp had not ached, would there then be a protrusion through or would you attempt to cap the pulp there?

Dr. Johnson. I think there would be sufficient to cause irritation there; it comes to the ragged edge.

Dr. Hunt. I think my testimony is a little like that of Dr. Wells; that there are a good many cases, in my treatment—and yet I feel as if there were so many cases that appear to be successes—that I should keep on capping nerves in what I consider favorable cases, and I would consider it a favorable case if we had accidentally exposed a nerve in such cases. I remember distinctly a circumstance and I doubted the man's statement, and yet he was a very intelligent gentleman. He was an old man and showed me a tooth with a very large amalgam filling in it. He had had a filling that he lost in crossing the Hudson River. He was a bridge-builder and fell into the hands of a man that thought it ought to be filled. It was a permanent molar and instead of filling it with amalgam he filled the tooth with gutta-percha, and he said he had had it in his mouth ever since, and when I looked at it, it was a healthy tooth and responded to the touch. I could hardly believe the statement, but he was an intelligent man and seemed to know all about it and remarked how he was saved from having an amalgam filling put in.

Dr. Chappell. We must bear in mind that whenever we consider tooth pulp exposed, the fibril may be irritated to that extent that irritation may affect a section of the tubules until there will be an exudation. We may have irritation sufficient extending down to the tubules and membrane and there will be effusion, and whenever that effusion takes place, that is to a certain extent an abscess. Whenever you put a capping on that, you have trouble with it sooner or later, just as certain as when you have an ulcerated membrane discharging through the canal of the tooth. Whenever you stop that discharge, septasenia sets in and trouble generally. Now whenever you have any indication there that there is effusion, that there is pulpitus, or anything of that kind leading to the effusion and discharge through those tubules, you must be very careful not to put any filling over there or to dam up these secretions that are being thrown off. Now there is the secret to a great many of those questions. There is another thing; there are a great many of those teeth that have begun to die at the same time the tooth had begun to decay. I mean the pulp has begun to die and recede. Sometimes the pulp begins after the period of ossification. That is a natural physiological effect. Then in the period of child-life, calcification sets in. Now, I would say to the students, all to remember, never to dam an abscess, or the abscess will "damn" you. In regard to antiseptic treatment of the wounds, you will use for anti septic dressing, bichloride dressing and bichloride preparations of cotton. Take a ball of cotton, put in a five hundred solution. This is as much bichloride as we can absorb in the treatment of all such cases, to fairly destroy the germs.

A Member. Supposing you are shot?

Dr. Chappell. Well, then, take five hundred and twenty-five per cent. You must provide all the time for taking up those exudations and stopping that condition. Sometimes we fail in spite of our work, and if we do allow it to pass off in that exudation, it will be taken back with irritation, and you know all the phenomena leading to inflammation, such as suppuration, ulceration, and so on. There may be a microscopical exudation there that we do not think of unless we are careful to take sufficient forethought to provide against it. If we fill a tooth of that kind, and place a dressing, reasonably or unreasonably large enough, after you have cleaned it out as well as you can, and washed it with warm water cooled to the temperature of the tooth, say ninety or ninety-eight, or whatever it may be, do not go beyond that unless you want to increase the exudation, if you run above the temperature of the tooth. Of course the temperature of the tooth is not equal to that of the soft parts, but then you must destroy the germs there, and then treat it as you would any other wound. Then you can put in a permanent capping and filling, and not until then, with any degree of certainty. But it differs now and then. I know that there are a great many cases where we destroy the nerve just as soon as we find any complaint, or pain, or anything of the kind. That is not necessary any more than it is to cut off a little piece of the nose because your nose bleeds. How many of you have seen pulp of teeth that has ruptured, and you have treated it, and after a few weeks you find that there is pulp remaining there, and there is a membrane grown there? The pulp has come out through an orifice, and strangulation takes place, and sometimes you make an application of pressure to bring it back to its natural condition again. You find the pulp, you go back to its place, and you can then cap it, and it will last for years with good success. Sometimes you can do that. Whenever you can not get it back, it will begin to inflame, and an abscess will form, and you will have your patient call again before taking it out. You must be guided by the conditions. Do not think we must always attempt the treatment of pulps, and capping of pulps, and saving teeth. We have patients who come into our offices and say: "I want that tooth saved; I want that pulp saved; I do not want any more abscesses." We have to do all that we can to save that pulp, and there is where our wits are tried, to save that pulp. It is an easy matter to save the crown, if they will keep the teeth clean. The question is to save the pulp. That is what we have to do, and if we do not do it we are to blame.

Dr. Morse. I would like to ask Dr. Chappell what his mode of capping the pulp is?

Dr. Chappell. The mode of treating the pulp?

Dr. Morse. Yes; capping the pulp when it is ready to put in?

Dr. Chappell. You come to the question that they have all asked. It is owing to the condition, as I said before. Molecules of the sulphuric acid and arsenic are migratory, and we are liable to have difficulty, but the least irritating substance you can get is gutta-percha. As I said awhile ago, burnish the walls of the cavity until you get to the center. Now, if you will use soapstone, as the Doctor said awhile ago, you can get that done, sometimes in certain cases using fine olive oil, and go over it, leaving a smooth surface. You can then build over that as a cap. I never want any pressure on it. I want that to be as soft as possible. In filling with gold, I do not want to fill the tooth with gold just then; I want to let the tooth rest. You can tell about the work whenever you see it; you know how it is done. I make it a rule whenever I turn out a patient to see a brother dentist and allow him to criticise; I want to do the very best work I can in that particular. I sometimes have patients in a preparatory state, or state of progress, that go into the hands of some other dentist who does not know how to do it; but as a general thing, your patients come back, and whenever you establish confidence in them, they will come back to you, and you will save the tooth. Do not be in too big a hurry about capping a tooth. After you place in the cotton and take out the exudation, whenever you are satisfied there is no effusion, then you can begin to put in the gutta-percha, if there has been great irritability there, and when you go to fill with gold it is only necessary for a surface. There is a good deal of difficulty in one respect, and this is in proportion as the tooth needs saving, gold is the meanest thing to try to save the tooth with, because you can not save it by putting a little cap over the top when there is effusion there, and there is a great deal of pathological disturbance. You can not save with gold, and you can not save with amalgam, and that is the reason why we have so many failures, because we are in too big a hurry. If a physician was to visit some of the brethren here, and give medicine with a view that that dose of medicine was going to make him well, so that he could come to the office the next day, you would be disappointed if he did not do it. We must not expect to treat a tooth and get it well in a minute; you can not do it, it can not be done.—*Indiana State Dental Association.*

